# The spatiotemporal prediction method of urban population density distribution through behaviour environment interaction agent model

**DOI:** 10.1038/s41598-023-32529-0

**Published:** 2023-04-10

**Authors:** Junyan Yang, Yi Shi, Yi Zheng, Zhonghu Zhang

**Affiliations:** 1 School of Architecture, Southeast University, 2nd Sipailou Street, Xuanwu District, Nanjing, 210096 China; 2 Southeast University Smart City Institute, Nanjing, China; 3 Research Centre for Chinese Nation Visual Image, Southeast University, Nanjing, China

**Keywords:** Environmental social sciences, Psychology and behaviour

## Abstract

Based on the interrelationship between the built environment and spatial–temporal distribution of population density, this paper proposes a method to predict the spatial–temporal distribution of urban population density using the depth residual network model (ResNet) of neural network. This study used the time-sharing data of mobile phone users provided by the China Mobile Communications Corporation to predict the time–space sequence of the steady-state distribution of population density. Firstly, 40 prediction databases were constructed according to the characteristics of built environment and the spatial–temporal distribution of population density. Thereafter, the depth residual model ResNet was used as the basic framework to construct the behaviour–environment agent model (BEM) for model training and prediction. Finally, the average percentage error index was used to evaluate the prediction results. The results revealed that the accuracy rate of prediction results reached 76.92% in the central urban area of the verification case. The proposed method can be applied to prevent urban public safety incidents and alleviate pandemics. Moreover, this method can be practically applied to enable the construction of a “smart city” for improving the efficient allocation of urban resources and traffic mobility.

## Introduction

With the progress of the Fourth Industrial Revolution, data science, digital tools, and information technology have made cities increasingly smarter. Based on this background, cities and urban society are newly characterized by a dynamic nature: short-cycle and high-frequency fluctuation^1^. Cities are gradually transforming into a smart complex system^2^. When the city is considered as a dynamic, complex, and smart system, there exist two researchable questions: understanding the law of its operation and making limited prediction in a certain spatial–temporal range based on the understanding of its operation law, in order to promote more efficient and reasonable operation of the urban system.

From the interdisciplinary research perspective of urban research and information science, the system operation of the entire city is considered as the complex interweaving and interaction of various “flows”^1^. Among them, human activities are undoubtedly the most important “flow” in the entire urban system. The spatial–temporal distribution and dynamic changes of human traffic have a significant impact on the functions, resources, and energy consumption of the city^3^. For the spatial–temporal dynamic distribution and flow law of people in cities, certain researchers argue that it follows a repeatable pattern within a certain spatial–temporal range^4,5^. The spatial–temporal distribution of the population is in line with the urban functions and is closely related to public demand. It reflects the behaviour orientation of people living in the city and the functions, resources, and facilities provided by the city to meet individual behaviour needs. Therefore, the characteristics and laws of population spatial–temporal distribution overlaps with the urban dynamic operation law to a certain extent^6–9^. In a city, the spatial–temporal distribution of people interacts with the urban built environment. The existing functions and infrastructure of a city affect the spatial–temporal behaviour of people. Simultaneously, the behaviour demands also cause corresponding changes in a city^10,11^. Accordingly, this paper argues that limited prediction can be realized by analysing the spatial–temporal distribution of urban population density.

Nevertheless, due to the complexity of the urban system itself, there are also arguments against whether the population can be predicted. Batty once highlighted that the ability to achieve accurate prediction was in fact severely limited due to population selection and behavioural complexity, although cities have their own internal laws that can be reflected through various “flows”^1^. He argued that even the role of prediction and the importance of prediction accuracy had basically disappeared. The study by Song on population movement model based on the data of anonymous mobile phone users expressed the limitations of prediction in human dynamics^12^. Moreover, human movement and activity patterns exhibited a considerable degree of freedom and variation, but they also showed structural laws owing to the knowledge of geographical and social factors^13^. Existing studies have also indicated that human activities are regular, predictable, and unique in time and space^12^. This study considers that it is difficult to fully predict people's spatial–temporal activities accurately in cities of different sizes and complexities^12^. However, based on the analysis of the law of population activities, it is still feasible to predict the spatial–temporal distribution of population density under limited conditions. For instance, Fan used Monte Carlo Markov Chain (MCMC) to cluster individuals with similar trajectories, and predicted the future behaviour of individuals based on the trajectory probability distribution of classified individuals^14^. Monreale used the T-mode tree to learn rules from trajectory patterns and predict individual behaviours^15^. There's no denying that predict the urban population density based on some extent is immense significance to improve the operation efficiency of an urban system. Meanwhile, conduct the accurate and dynamic prediction of urban population design is also important for prevent the occurrence of public safety incidents, such as stampede, and is also can provide necessary support for city traffic management and complete the construction of smart cities^16,17^.

## Critical review of four main prediction methods

In general, the spatial–temporal distribution of urban population density is predicted by mining the dependency laws of the population in the two dimensions of urban space and time^13^. Then, we can predict the population density distribution in an unknown time span and spatial range by learning and mastering the dependency laws of this relative steady state^18^. According to different basic theories and methods, there are four major methods existing for predicting urban spatial–temporal behaviours ^19–21^.

### Methods for population activity prediction based on prior theory

These research approaches, proposed based on hypothesis theory, first realize the real approximation of the prediction results by constructing logically appropriate parameters and models and then understand the impact of various factors on the population density distribution by explaining such parameters and models^22,23^, for example, estimation of the mobility based on the gravity model and prediction of the spatial–temporal distribution density of the population. In terms of characteristics, such studies mainly make a macro summary of the distribution of population density from a theoretical perspective, build relevant hypotheses from the top to the bottom according to the prior theory and find appropriate parameters through observation data, so the prediction results are better explanatory. However, in terms of the results, such models can only be used as an ideal approximate model to describe the distribution of urban population density, but cannot accurately locate specific space units. Therefore, the spatial distribution of dynamic population density cannot be reflected accurately^10,24,25^.

### Methods for population activity prediction based on statistics

It can be found that human travel follows a simple and repeatable pattern based on the statistics of a large number of individual behaviours mainly through mathematical statistics. Furthermore, a specific statistical model is used to evaluate the relationship between various influencing factors and the actual population density distribution, so as to predict the population spatial–temporal distribution^26–29^, Moreover, such methods can capture the trend, periodicity and other characteristics in the time series, but such models cannot describe the spatial impact in urban population density prediction and is often used in the field of such short-term population density estimation as traffic flow estimation^30,31^.

### Methods for population activity prediction based on dynamics

Based on the transfer law of micro individuals, a state framework of system macro behaviours has been established as a new perspective of urban population distribution prediction. It brings the decision-making and transfer of micro agents into the scope of modelling, and tries to explain the impact and changes of local interaction on the overall system by establishing a transfer function of its own state and domain state^32–34^. However, the complexity and confusion of population activities are relatively higher, so the simple and subjective conversion rules based on the mathematical statistical model will oversimplify the whole dynamic process^35^. Furthermore, the controllability of the model is poor, and the model test is more difficult on the micro scale^36,37^.

### Methods for population activity prediction based on machine learning

It uses the growing spatial–temporal data flow as a learning database, from which it tends to a fixed population activity pattern in advance. Furthermore, this model can be applied to predict the population density distribution effectively in a certain space–time range in the future^38^. This method includes two branches: one is the population distribution prediction algorithm based on the traditional machine learning models such as support vector machine, Gaussian and Bayesian models. Chen used the Inhomogeneous Gaussian Markov Random Field (IGMRF) model to predict the regular changes of human traffic^39^. The other branch is the algorithm which uses a processing layer composed of multiple nonlinear transformations to carry out high-level abstraction of data^40,41^ in the field of deep learning at the forefront of machine learning, for example, the ST-ResNet residual convolution model and the LSTM cyclic neural network model are used to predict the regional human traffic^42,43^.

Through reviews the above researches, it can be found that current studies have major studied the behaviour patterns of crowds through modelling and algorithms. The advantage is that they can objectively analyse the behavioural patterns of crowds themselves. Afterwards, it provides the necessary methodology support for the prediction of the urban population spatial and temporal distribution based on behavioural rules. However, the general limitation of the current researches is that the coupling mechanism between population behaviour and urban environment has not been established, so as to correlate the two main variables of crowd behaviour and environmental characteristics to predict the spatial and temporal distribution of urban population density. Therefore, in this research the interaction between population behaviour and environmental characteristics is considered as the basic logic to predict the spatial–temporal distribution of population density in a certain range in the future. Furthermore, the behaviour–environment agent model is established, and the depth residual network model in the neural network is used to conduct limited prediction of the spatial–temporal distribution of the urban population density.

## Interrelationship between behaviour and environment

### Interrelationship between urban built environment and population spatial–temporal behaviours

**Figure 1:**
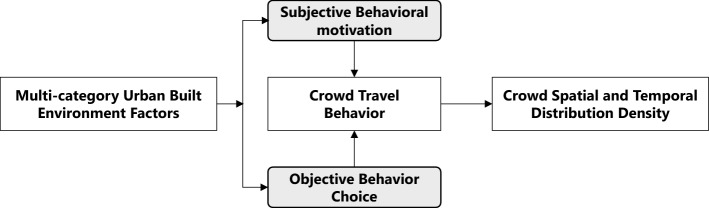
Brief concept model of interrelationship between behaviour and environment. *Source* Author made.

In a city, the population spatial–temporal activities are usually determined by travel motivation and preference. Among them, travel motivation mainly depends on the unique needs of individuals, so it is difficult to summarize it as a universal pattern. Travel preference mainly reflects the behaviour choices made by individuals in the process of travel under the dual effects of external objective environmental factors and subjective preferences^44,45^. According to Maslow's hierarchy of needs, people consider the basic needs of the objective environment in terms of safety, accessibility and convenience when making travel choices, as well as the satisfaction of functional, social and service requirements^46,47^. The results of the existing research on population behaviours in Anthropology and Social Sciences indicate that people of similar age, social class and educational background usually have similar travel choice behaviour^48^. Moreover, the urban objective environment can meet people’s travel needs and also has certain constraints on their travel behaviours^49^, so that under the impact of urban built environment, the population spatial–temporal behaviours will show regular characteristics and a regular trend. Such a law under the interaction between urban built environment and human activities has also become the main basis for predicting the spatial–temporal distribution of urban population density (Fig. 1).

In terms of the influencing factors of the built environment and people's spatial–temporal behaviours, a large number of existing studies have shown that spatial location, land function, spatial capacity, traffic facilities and business services have a major impact on the agglomeration and activities of people in cities^50,51^. Miranda discussed the relationship between the characteristics of urban built environment and the spatial experience of people by analysing pedestrian trajectories based on GPS data, and pointed out the impact of public facilities and quality in the built environment on people's activities at the level of stay time^52^. The study of Limtanakool found that even considering the impact of individual socio-economic characteristics and travel time, the urban land function is still the basic factor that determines people’s travel behaviours^21^. On the basis of land function, the urban business form plays a more decisive role in the stay time and activity intensity of people. To a large extent, the type of land use determines the type of people’s activities. The richness and diversity of urban business forms and the degree of fit with the behavioural needs of the population directly affect the aggregation and dispersion of the spatial–temporal distribution of population density^53^. Moreover, in addition to the relevant indicators at the neighbourhood level, the urban form and traffic network types at the regional level also have a certain impact on residents' behaviours in work, shopping, recreation and leisure travel^54,55^.

In terms of the impact characteristics of the built environment on people’s spatial–temporal behaviours, the impact of the built environment on human behaviours basically conforms to the laws of geography. Based on the First Law of Geography, the inflow and outflow of people in adjacent areas affect each other and people’s activities are spatially dependent^56^. In terms of the built environment, the built environment in adjacent areas is more correlated than that in remote areas as a whole^56,57^. Areas with similar built environment are similar in population activities. However, at the same time, the impact of built environment on spatial–temporal behaviours are somewhat complex and will specifically impact the spatial and behavioural heterogeneity at the level of results^58^. This means that even if different built environments have similar environmental characteristics, people’s activities may follow different laws^59,60^.

### Key impact factors of urban built environment on population spatial–temporal distribution

Based on the interaction between the urban built environment and the spatial–temporal behaviour of the people, the impacts of urban built environment on people's spatial–temporal behaviours can be classified in five dimensions: spatial location, land use structure, spatial capacity, traffic facilities and functional business forms^19,48,49,59,63^. Accordingly, such five impact dimensions can be further classified according to their impact paths and modes in the actual interrelationship, finally to form an indicator system for the impact of urban built environment on the spatial–temporal distribution of people, including 5 major categories, 10 medium categories, and 40 small categories (Table 1). Such an indicator system can be applied as the setting of spatial basic conditions for limited prediction of the spatial–temporal distribution of urban population.

**Table 1:** List of impact indicators of urban built environment on population spatial–temporal distribution. *Source* Author made.

Major category	Medium category	S/N	Small category	Label code	Paraphrase
Spatial location	Central location	1	Distance from the main center	dia	The spatial location refers to the location of the target space of human behaviour in the city. Under normal conditions, the distribution density of urban population decreases with the increase of distance from the center^59,61^
		2	Distance from the nearest subcenter	Dib	
		3	Distance from Level-3 centers	diC	
	Traffic location	4	Distance from junction station	hub	
Land use structure	Function type	5	Public service management and service land use proportion	A	The land use structure refers to the proportion of various types of land in a certain space of the urban built environment and its interrelationship; as an element that has a direct impact on people’s travel behaviours, it can reflect people’s different travel purposes, such as education and medical care^62^
		6	Proportion of land for colleges and universities	A31	
		7	Proportion of land for primary education	A33	
		8	Proportion of land for medical treatment and public health	A5	
		9	Proportion of land for commercial service facilities	B	
		10	Proportion of land for special use	D	
		11	Proportion of land unfit for construction	E	
		12	Proportion of village land	E6	
		13	Proportion of park green land	G1	
		14	Proportion of protective green land	G2	
		15	Proportion of square land	G3	
		16	Proportion of land under construction	K	
		17	Proportion of industrial land	M	
		18	Proportion of residential land	R	
		19	Proportion of traffic land	S	
		20	Proportion of municipal land	U	
		21	Proportion of land for logistics storage	W	
		22	Proportion of road land	ROAD	
	Land function structure	23	Land use information entropy	landuse_q	
		24	Land use balance	landuse_j	
Space capacity	Development density	25	Building density	Density	The spatial capacity mainly determines the carrying capacity of the behavior target space to the population activities; in general, the distribution of population density is directly proportional to the development intensity of the land^63^
	Development intensity	26	Plot ratio	Far	
Traffic facilities	Road traffic	27	Road network length	Road length	The traffic facilities as important spatial carriers of population flow have a direct impact on the number of residents' trips and the choice of travel modes. Simultaneously, different traffic facilities also determine the accessibility of people's travel motivation and the specific means of transportation selected according to different travel motives^59,64^
		28	Highest road grade	Road rank	
		29	Topological depth	Topo depth	
	Public transport	30	Distance from subway station	Metro	
		31	Number of metro stations	Metro Count	
		32	Number of bus stations	bus Count	
	Other traffic facilities	33	Railway length	Rail length	
		34	Number of railway stations	Rail stat	
		35	Number of airports	Airport	
		36	Number of airport passageways	Air entry	
Functional business type	Number of business type functions	37	Number of social service function POI points	Social service	The functional business forms mainly based on POI data (point of interest data) with fine granularity reflect the aggregation status of various functions in space^62^
		38	Number of life service function POI points	Life service	
		39	Number of production service function POI points	Production	
		40	Number of industrial manufacturing function POI points	Industrial	

The above indicator system is mainly defined based on the impact of built environmental factors on the motivation and choice of people's travel behaviours. Inclusively, in terms of the impact of the built environment on the subjective motivation of people's travel behaviours, the impact of the built environment is mainly reflected in whether it can trigger people's travel motivation for a certain area in the city, that is, whether the area is attractive for people’s travel. From the urban spatial perspective, the impact indicators mainly include spatial location, land use structure and spatial capacity. Moreover, the impact of the built environment on people's travel choice is mainly reflected in whether the target space can meet people's travel motivation and whether the relevant functions can be easily accessible. In this regard, it mainly includes such two sub-indicator systems as traffic facilities and functional business types. In the functional business types aspect, the number of POI points within a certain spatial range are directly related to the satisfaction of people's travel motivation, and will directly affect people's choice of travel destination, thus affecting the distribution of population space–time density^62^.

### Prediction model framework of population space–time density based on built environment

**Figure 2:**
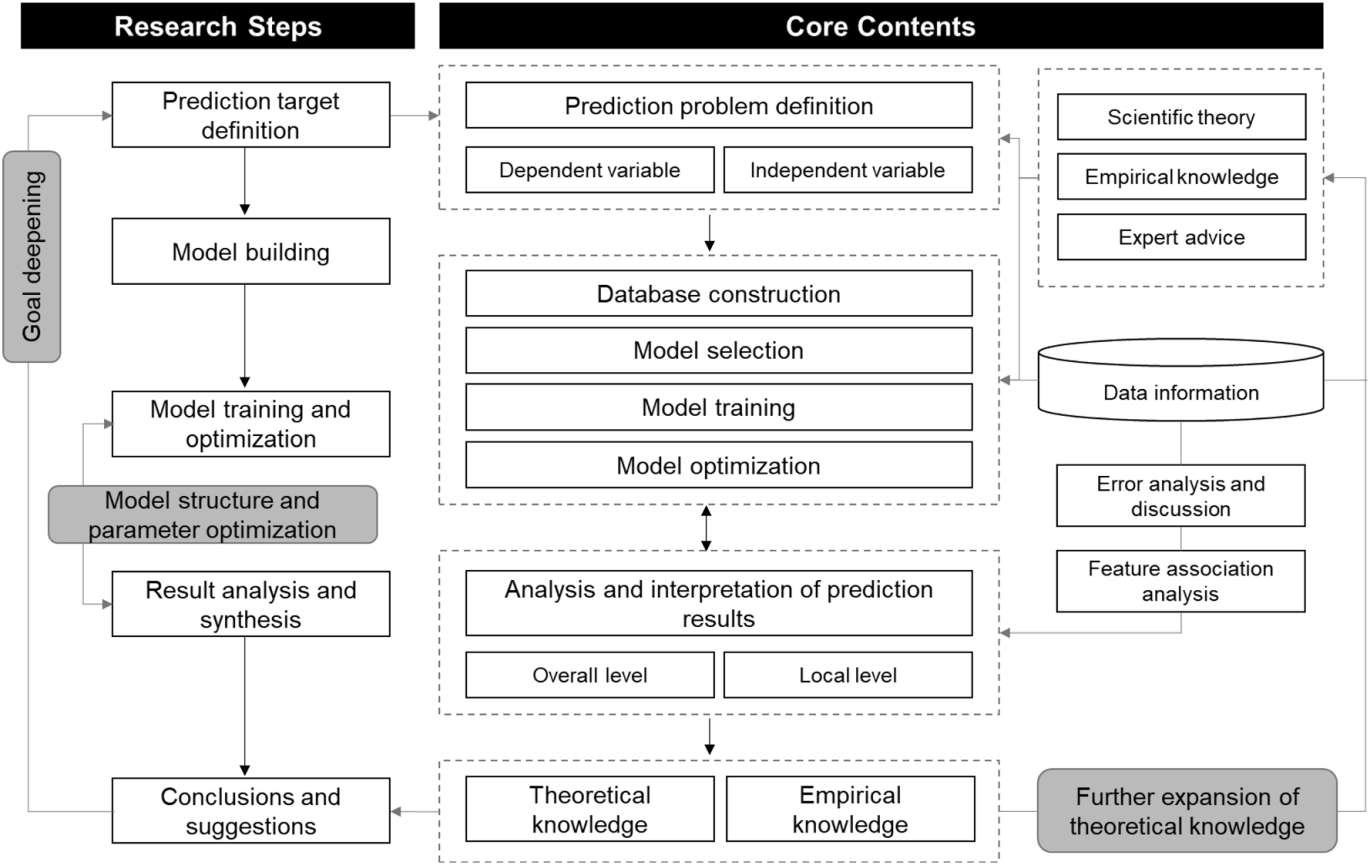
Prediction model framework of spatial–temporal crowd density. *Source* Author made.

Based on the influencing factor indicators and the general laws of people's activities in urban built environment, the data-driven prediction model framework of population space–time density in built environment is summarized and established (Fig. 2). It is worth noting that the framework is not a simple linear structure, but emphasizes the idea of system and iteration, which means that the analysis of results may provide suggestions for further optimization and theoretical interpretation of the model. While solving actual problems, we may adjust various parameters of the model constructed in the third step according to the preliminary prediction results obtained in the fourth step; or, on the basis of the prediction results, we can further define the problem combined with the theoretical knowledge or empirical tests that have not been taken into consideration before.

#### Construction of prediction model

On the basis of urban multi-source big data acquisition, determine the data pre-processing method, build a database that can accurately match the problem, mine the characteristic variables, and select the appropriate model framework. Here the residual network model is selected as the modelling method to fit the theoretical basis and laws of urban planning and environmental behaviour. Subsequently, construct a feature data set of population density spatial–temporal distribution and built environment to provide the basis for model training and optimization.

#### Model training and optimization

Input the feature processed data in the database into the model; iterate and optimize the model through system simulation or model training. Introduce the concept of feedback loop in biology and subsequently, obtain a part of the system output as input, such that the system could adjust its performance to meet the required output; for example, in machine learning, use iteration or batch training to adjust hyperparameters and optimize the model.

#### Result analysis and synthesis

The analysis and synthesis of prediction results includes the core contents as follows: (a) Globally, evaluate the model from prediction accuracy, summarize the regularity of the prediction accuracy distribution and the applicability scenarios of the model, and use the planned and agreed standardization theory to understand certain characteristics of the model. (b) Partially, group the prediction objects according to their basic characteristics and perform classified interpretation. When the model fails to meet the prediction expectations, we can classify the prediction errors, explore the laws or potential impact factors reflected by the errors based on field investigation and statistical analysis, and further summarize and conclude the application boundaries and scenarios of the prediction model, in order to achieve a deep understanding of the model.

#### Practical application and deployment

The optimized model can be deployed for urban public safety monitoring, population activity trend judgment, major event management, epidemic prevention, and other relevant application scenarios involving population space–time activities and distribution laws, in order to serve as a part of the urban decision support system and provide action plans for urban decision-making.

## Construction of BEM prediction model

### Method for construction of BEM prediction model

According to the prediction model framework of population spatial–temporal density proposed above, the current study converts the population spatial–temporal distribution and built environment characteristics into images and the sequential prediction problem into a graph prediction problem. Thus, the convolution neural network method can be effectively used. For the construction of population spatial–temporal distribution model, the convolutional neural network method has certain advantages as follows: (a) Convolution and pooling layers are used, such that the feature factors can be automatically extracted from the built environment, avoiding the error caused by the manual selection of feature factors. (b) The operation method of convolution layers can effectively solve the problem of spatial correlation and incorporate spatial autocorrelation within the modelling scope. Moreover, the pooling layer can significantly improve the operation efficiency of the model and be extended to large-scale problems in cities. Considering the problems of overfitting and gradient disappearance with the deepening of network layers in deep learning, the deep residual network ResNet in convolutional neural network is finally applied to the model construction in this study^65,66^.

The deep residual network ResNet uses the concept of cross layer linking, and the problem of accuracy reduction with the deepening of network layers can be better solved via shortcut linking.

The model considers the built environmental factor as the independent variable and the time–space distribution of the population on the working day (population density) as the dependent variable. Firstly, construct the feature data set of population multi-period density and build environmental factors; secondly, input the standardized data into the model; finally, iterate and optimize the model through model training. The model construction includes the following four steps: data pair processing and construction, data set construction and division, model construction, and model training and output. A database containing 40 built environmental factors and the spatial–temporal distribution characteristics of population density were constructed, and the deep residual network ResNet was selected to construct a convolutional neural network model to simulate the spatial–temporal distribution of population in the case city^67^.

### Data processing and input

For the spatial–temporal distribution data set of population density, the prediction targets a 24-h expected distribution in the form of 24 time series which are not completely independent. The output labels need to be processed considering the correlation of the time series data. However, for the built environment data set, in order to render all features mutually comparable and consider the spatial correlation of the built environment, it is also necessary to process the features of the built environment, in order to build a prediction label data pair that is conducive to model training.

#### Processing of population spatial–temporal density data

**Figure 3:**
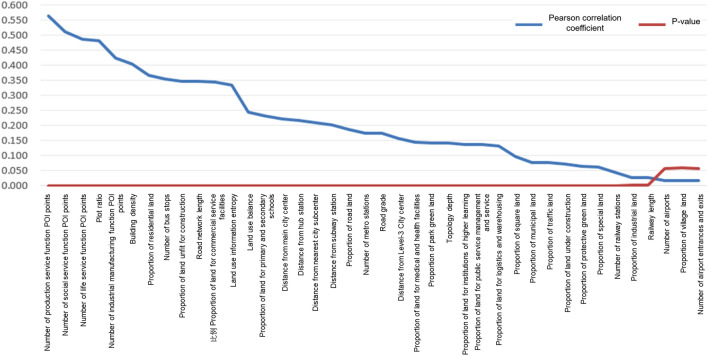
Eigenvalue correlation score and p_value. *Source* Author made.

For each spatial analysis unit k, we need to predict the spatial–temporal distribution sequence of population density with the objective of specifying any grid,

$${\text{Y}}_{i,j}^{k} ,{\text{Y}}_{i,j}^{k} = \left\{ {y_{i,j}^{k} [0],y_{i,j}^{k} [1],y_{i,j}^{k} [2], \ldots ,y_{i,j}^{k} [23]} \right\}$$

while ${\text{Y}}_{i,j}^{k} [{\text{t}}]$ is the evolution label of the spatial–temporal density of the kth grid at time t. The principal component analysis method is used to reduce the dimension of the 24-h population distribution; ${N}_{prim}$ indicates the number of extracted principal components for model training for reducing the dimension and improving the efficiency. In the process of model output, the evolution results of the 24-h population density can be obtained again by inverse transformation of the principal components. After calculation, when ${N}_{prim}$ = 6, the principal components can explain 98.7% of the variance; therefore, the first six principal components ranked in a descending order according to the variance proportion after dimensionality reduction by principal component analysis are considered as the feature labels (Fig. 3), and the output label of the spatial–temporal distribution of population density after dimensionality reduction is recorded as ${y}^{PCA}$.

#### Processing of built environment feature data

Based on the system (as mentioned in Table 1) for 40 types of impact indicators of the built environment, the Pearson correlation coefficient can be used to select the features. Simultaneously, the Pearson coefficient needs to be modified according to the data features used for prediction in the study. The modification considers the important factors in the label features *y*^*PCA*^ after dimension reduction. As long as the built environment features exhibit one-dimensional correlation (the absolute value of the correlation is greater than 0.1) in the extracted *N*_*prim*_ factor values after dimension reduction of the principal component and the p_value can pass the significance test of 0.05, such features can be included within the scope of training. The Pearson correlation coefficient modified has such a formula as follows:

1 $$p_{j} = \mathop {\max }\limits_{{k \in \left\{ {1,2, \ldots ,N_{prim} } \right\}}} \sum\limits_{i = 1}^{n} {\left( {\left| {\frac{1}{n - 1}\sum\limits_{i = 1}^{n} {\left( {\frac{{X_{ij} - \overline{{X_{j} }} }}{{S_{{X_{i} }} }}} \right)\left( {\frac{{Y_{ik} - \overline{{Y_{kj} }} }}{{S_{{Y_{k} }} }}} \right)} } \right|} \right)}$$

where *X*_*ij*_ represents the normalized characteristic value of the jth feature in the ith sample, $\overline{{X_{j} }}$ represents the normalized mean value of the jth feature, $Y_{ik}$ represents the value of the kth principal component in the ith sample on the sample, $\overline{{{\text{Y}}_{{{\text{kj}}}} }}$ represents the mean value of the factor value of the jth principal component on all samples, $S_{{X_{j} }}$ represents the standard deviation of the jth feature, and $S_{{Y_{k} }}$ represents the standard deviation of the factor value of the kth principal component on all samples. *N*_*prim*_ represents the number of the principal components obtained via dimension reduction by the principal component method in the previous step. For the prediction target task of the study, irrespective of whether the correlation is positive or negative, it is beneficial to model training. Therefore, the modified Pearson coefficient assumes its absolute value. The closer *p*_*j*_ is to 1, the greater the correlation between the population spatial–temporal feature label and the built environment label data, but the closer it is to 0, the smaller the correlation. Furthermore, we calculate the Z statistic value for the Pearson correlation coefficient of each feature, and subsequently obtain p_value and set p = 0.05 as the significance level. It shows the feature correlation scores and p-value values arranged in a descending order based on the *p*_*j*_ value. For the 40 input variables, 37 variables have their Pearson coefficients pass the correlation test, and the correlation coefficients of the first 32 variables greater than 0.1 are selected and retained.

#### Construction results of feature data pairs

Based on the above-mentioned characteristics of population space–time density and built environment, we can obtain the input feature pairs (*x, y*^*PCA*^) in a uniform format. For each spatial analysis unit i, the input built environment feature *X*_*i*_ is a three-dimensional array: the first dimension represents the built environment feature of the spatial analysis ontology, the second and third dimensions represent the plane distribution of the features of the space unit and its surrounding built environment and represent the data of the spatial analysis unit and its adjacent (2h + 1)^2^ − 1 spatial analysis units; h = 5 is selected in this study. *y*^*pca*^ is the weight of the principal component after dimension reduction. The training data pair is (*x, y*^*pca*^). For the training result *y*^*pca*^, the 24-h population evolution prediction result Y = {y[0], y[1], y[2],…,$\mathrm{y}[23]\}$ can be obtained by PCA inverse transformation.

#### Data set filtering and division

**Figure 4:**
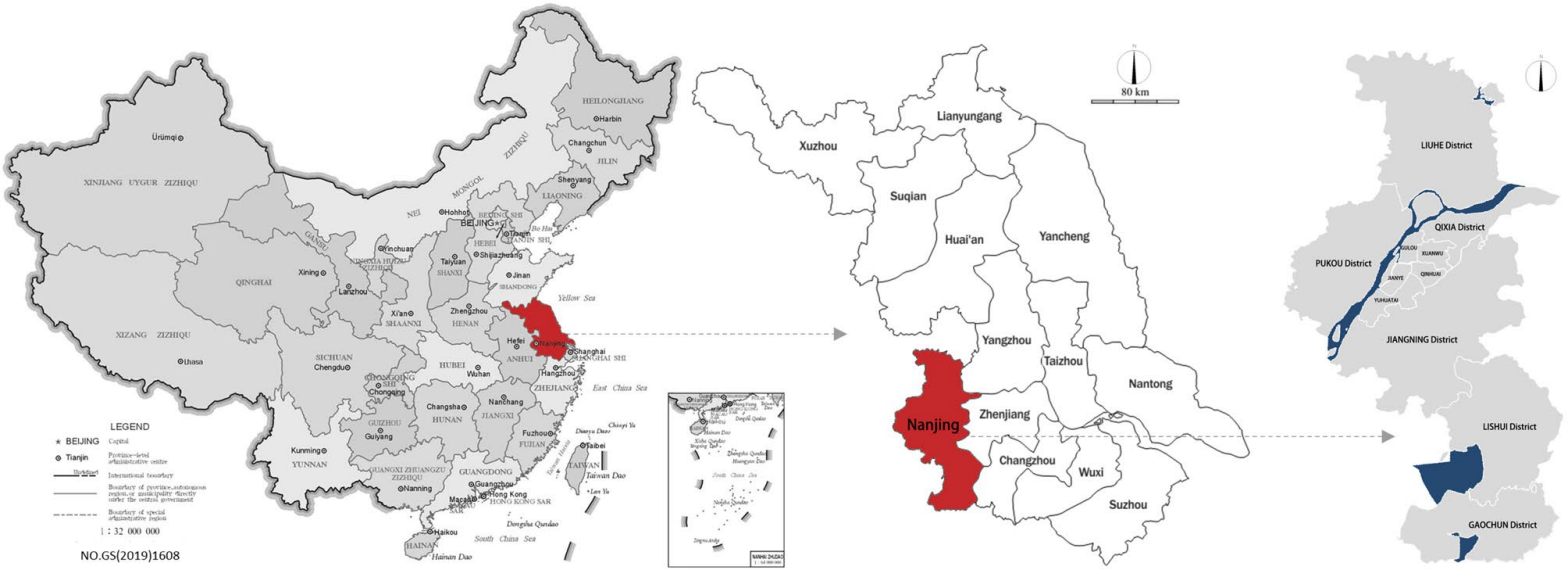
Location of the city of study in China’s map. *Source* Author made through tianditu JavaScript API 4.0 open access platform (http://lbs.tianditu.gov.cn/api/js4.0/opensource/source.html).

**Figure 5:**
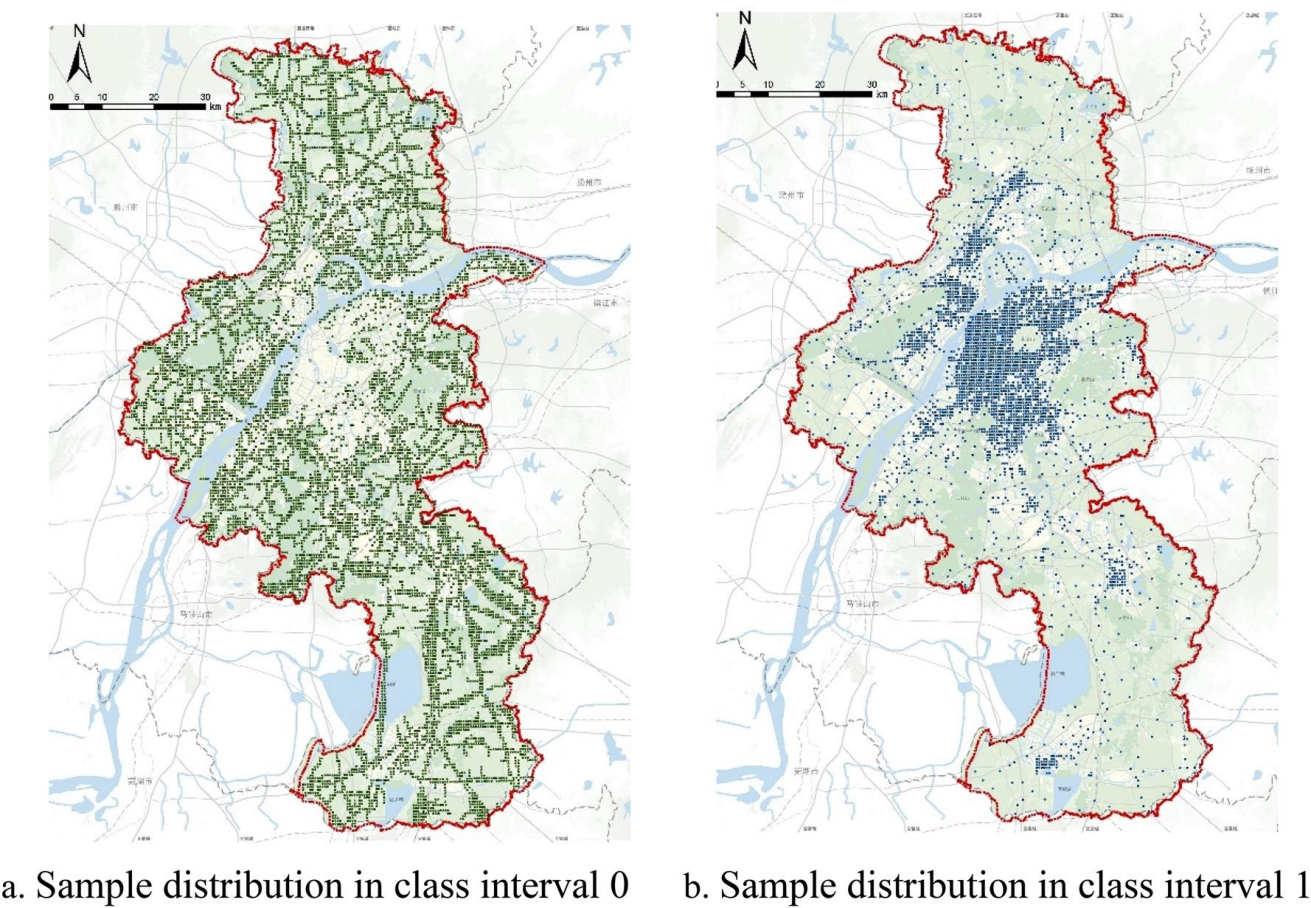
Sample distribution of classified training data set. *Source* Author made based on ESRI ArcGIS Pro Software (https://pro.arcgis.com/en/pro-app/latest/get-started/get-started.htm).

Based on the urban actual situation and the characteristics of simulation methods, the data set can be further filtered and divided. The average distribution of population density presents power law characteristics. 20% of the spatial grids account for more than 80% of the human traffic, while the remaining 80% of the grid land is almost completely the land unfit for construction. There are 9210 space units with a population of less than 200, accounting for 73.56%. If all the analysis units are directly applied to data model training, the model training results may be seriously inclined to the space units with fewer people, resulting in large errors. Therefore, in this paper, the classification targeted learning method is adopted to divide the data samples into two categories for classified training. The one with the actual daily average human traffic less than 200 is recorded as the class interval 0, while the one more than 200 is recorded as the class interval 1. Most of the spatial analysis units with a daily average population less than 200 people are distributed in the suburban areas, while those with a daily average population more than 200 are distributed in the central urban area (Figs. 4, 5). Through this step, the possible interference from a large number of LUFC areas in the city to the model calculation results can be eliminated. Furthermore, the accuracy of finite prediction can be improved under certain conditions.

### Model construction choice

**Figure 6:**
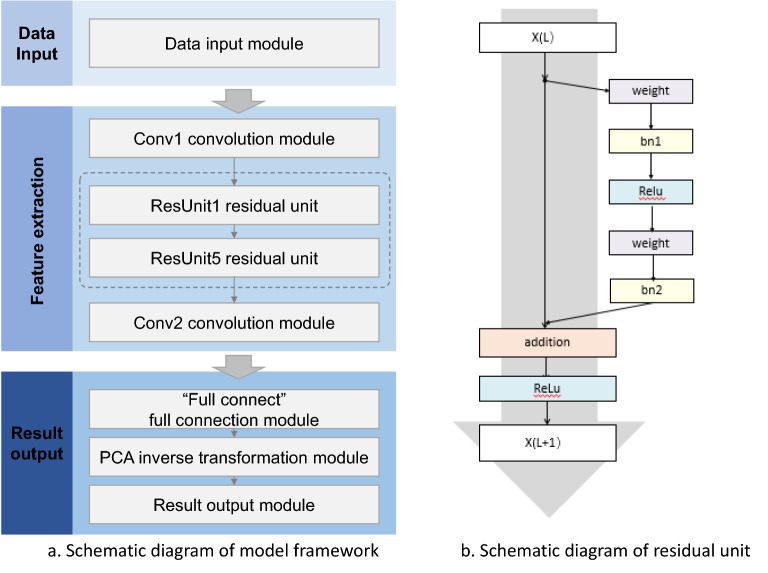
Schematic diagram of model construction framework and residual unit structure. *Source* Author made.

According to the advantages of the convolutional neural network method in automatic extraction of built environment feature factors, model construction, and prediction efficiency, the depth residual model ResNet in the convolutional neural network (CNN) model is selected as the basic modelling framework. The convolutional neural network (CNN) has shown a strong ability of capturing spatial structure information hierarchically, while the residual units can increase the depth of neural networks and reduce gradient loss^67^. The basic modelling framework consists of three steps: data input, feature extraction, and result output (Fig. 6). The method of batch training is used to update the parameters iteratively. First, input a small batch of training samples in the training set to obtain the simulation results through multi-layer convolution. Thereafter, use the obtained error, and for each batch of data input to the neural network, input the loss function score into the error function; subsequently, use the adam optimizer to determine the gradient vector by inverse derivation and adjust each parameter in the network according to the gradient vector to cause the error to converge and ultimately optimize the hyperparameters. After all batches are updated, input the validation set into the model to obtain the simulation effect of the model on the validation set and subsequently calculate the root mean square error between the actual 24-dimensional results and the simulated 24-dimensional results. This process is called an iteration cycle. In subsequent iteration, use the “Early Stopping” method to determine the number of iterations and consider the model parameter corresponding to the historical minimum root mean square error of the validation set as the final result.

The data input module in Step 1 is used to read the pre-processed feature data pair (*x, y*^*pca*^), and the step feature extraction refers to the calculation and extraction of existing features using multi-layer convolution to obtain a feature set with a higher degree of abstraction. The convolution layer module is used to expand and pool the input features; the residual layer module includes continuous residual units for deepening the network depth. For the residual network module, since the input-built environment feature image is smaller than those in the field of traditional image recognition, set the number of residual units in this model as 5 considering the built environment feature size and calculation time. In Step 3, the full connection layer module is used to map the distributed features learned by the convolution and residual modules to the number of reduction dimensions *N*_*prim*_ of the sample label space to PCA; the PCA inverse transform module is used for inverse transformation of the result after dimension reduction, in order to obtain a 24-dimensional vector of the population density changing with time in the problem definition; the data output module is used to output the prediction result. Simultaneously, the urban grid data is divided into training set, validation set, and test set according to the ratio of 8:1:1. The training set is used to train the model, the validation set is used to check the training degree of the model for parameter adjustment and model selection, and the test set is used to test the final effect of the model and evaluate the accuracy, applicability, and error of the model. The data set is divided by random sampling and the division is relatively well-distributed; therefore, excessive concentration or partial loss can be avoided.

## Model evaluation and error possibility analysis

After the prediction model has been constructed and trained, a certain scale of test sets is used to evaluate the performance of the established prediction model. For the evaluation of the population spatial–temporal distribution density prediction model, the core indicator is the prediction error based on the test set, and the smaller error indicates higher prediction performance of the model.

### Model efficiency evaluation index and algorithm

In order to evaluate the scientificity of the convolution neural network method based on the residual network adopted by the model, three algorithms, namely, the multiple linear regression algorithm, the XGBoost algorithm based on the decision tree, and the convolution neural network algorithm (CNN) without residual units are adopted to calculate the data set and compare the results. According to the average absolute error (MAE), root-mean-square error (RMSE), and hit rate (Precision-a), the convolution neural network model based on the residual network adopted in this study is better in terms of accuracy of fitting^68^.

Considering that the research data set is characterized by a large sample size, severe data fluctuation, and uneven data distribution, it is necessary to adjust the indicators of measurement errors. For the evaluation of the overall performance of the model, root-mean-square error (RMSE) is suitable for comparing the errors between the simulated results and the actual results of the data set, but not for comparing those data sets different in magnitude. Therefore, the normal root-mean-square error (NRMSE) which can lower dimensional effects is used to comprehensively evaluate the overall performance of the model; the definition is as follows:

$${\text{NRMSE}} = \frac{RMSE}{{MAX(s_{train} ) - MIN(s_{train} )}},$$

$${\text{RMSE}} = \sqrt {MSE} = \sqrt {\frac{{\sum\nolimits_{{(x,y,y^{24} ) \in S_{test} }} {\sum\nolimits_{i = 0}^{23} {(f(x)_{i} - y_{i} )^{2} } } }}{{\# (S_{test} *24)}}} ,$$

where$f(x)_{i}$ refers to the predicted population density at the ith moment after the environmental feature vector x is established for one grid input feature, $y_{i}$ represents the actual population density at the ith moment, and $\# (S_{test} )$ represents the number of elements in the test set.

Average absolute error (MAE) refers to the absolute average value of the error between the observed value and the real value which can avoid the problem of the positive and negative errors cancelling each other; the definition is as follows:

$${\text{MAE}}_{k} = \frac{1}{24}\sum\limits_{i = 0}^{23} {\left| {f(x)_{i}^{k} - y_{i}^{k} } \right|,}$$

where $f(x)_{i}^{k}$ indicates the predicted value of the population density of the space unit k at time i, while $y_{i}^{k}$ indicates the actual value of the space unit k at time i.

Average absolute percentage error (MAPE) features the advantage of being independent of proportion; therefore, it is often used to compare the prediction performance of different data sets. However, owing to the imbalance of data, it causes serious deflection. In a plot less crowded, if the MAPE index is adopted, MAPE increases because of the smaller denominator. For the case city, however, the less crowded space units account for more than 70% of the total. If all follow this indicator, the overall MAPE will be rather large. Therefore, we can optimize the basic MAPE and subsequently calculate the MAPE indicator at different times according to the actual situation of the population density. The segmented MAPE is defined as follows:

$${\text{MAPE}}_{j} = \frac{1}{{\# (S_{{test_{j} }} )*24}}\sum\limits_{{(x,y,y^{24} ) \in S_{{test_{j} }} }} {\sum\limits_{i = 0}^{23} {\frac{{\left| {f(x)_{i} - y_{i} } \right|}}{{y_{i} }}} } *100\% .$$

Furthermore, because there is a certain error between the predicted value and the real value, the concept of prediction accuracy is introduced in this paper to evaluate the model results. It represents the proportion of the number of samples whose average absolute percentage error is less than or equal to A*yi to the total number of samples. The larger the Precision – *a* value, the more the number of predicted samples accounts for the total number of samples for the set ratio. The accuracy rate is defined as follows:

$${\text{Precision}} - a = \frac{{\sum {\text{Sgn}}\left( {{\text{MAPE}}_{j} \le a} \right)}}{N}*100\% ,$$

where sng (x) represents the indicator function, and it returns 1 when x is true, but 0 when x is false; *a* represents the fluctuation ratio, while N represents the total number of samples. The larger the Precision – *a* value, the higher the prediction accuracy of the model under the set *a* fluctuation ratio. In this paper, $a$ = 0.3 is considered.

See the following table (Table 2) for the final calculation results predicted by the algorithm on the data of the test set and the comparison of the evaluation results of each index.

**Table 2:** Comparison of evaluation indexes of simulation results of various algorithms. *Source* Author made.

Calculation method	MAE	NRMSE	Precision-a
Multiple linear regression	213	0.0274	11.02%
XGboost algorithm	135.95	0.0231	34.50%
Convolutional neural network algorithm (CNN) without residual elements	147.24	0.0245	35.40%
Convolutional neural network model based on residual network	132.42	0.0227	37.80%

### Model error distribution analysis

As can be seen from the model calculation results, the mean absolute percentage error (MAPE) in each crowded area shows a power-law distribution with the increase in the population density. After the daily/hourly average number of people in the research unit exceeds 800, the MAPE remains stable below 30%, but the average percentage error is relatively high in the space unit with the daily/hourly average number of people below 800, indicating that the model is more adaptable to the highly crowded plot. Moreover, the plot less crowded has a larger MAPE, but the population density base is low; thus, the absolute value of model deviation is low. On this basis, we can compare the MAPE of the core, central, main, and overall urban built spaces (Table 3). In the core-built space, the research units with MAPE less than 30% account for 76.92%; thus, the model fitting effect is appropriate. For the space units other than the core built space, the MAPE increases significantly with the expansion of the central built space, main built space, and overall space circle, indicating that the accuracy of the model fitting decreases with the expansion of the urban circle.

**Table 3:** Distribution proportion of MAPE in different space areas. *Source* Author made.

Spatial circle	0–30%	30–50%	50–100%	Above 100%	MAPE
Core built space	76.92%	7.69%	15.39%	0%	27.18%
Central built space	30.60%	20.30%	11.70%	37.30%	97.23%
Mainbuilt space	25.41%	21.08%	17.73%	35.67%	141.54%
Overall urban space	23.20%	15.60%	25.20%	35.67%	233.80%
MAPE (mean absolute percentage error) distribution in each population density interval					

**Figure 7:**
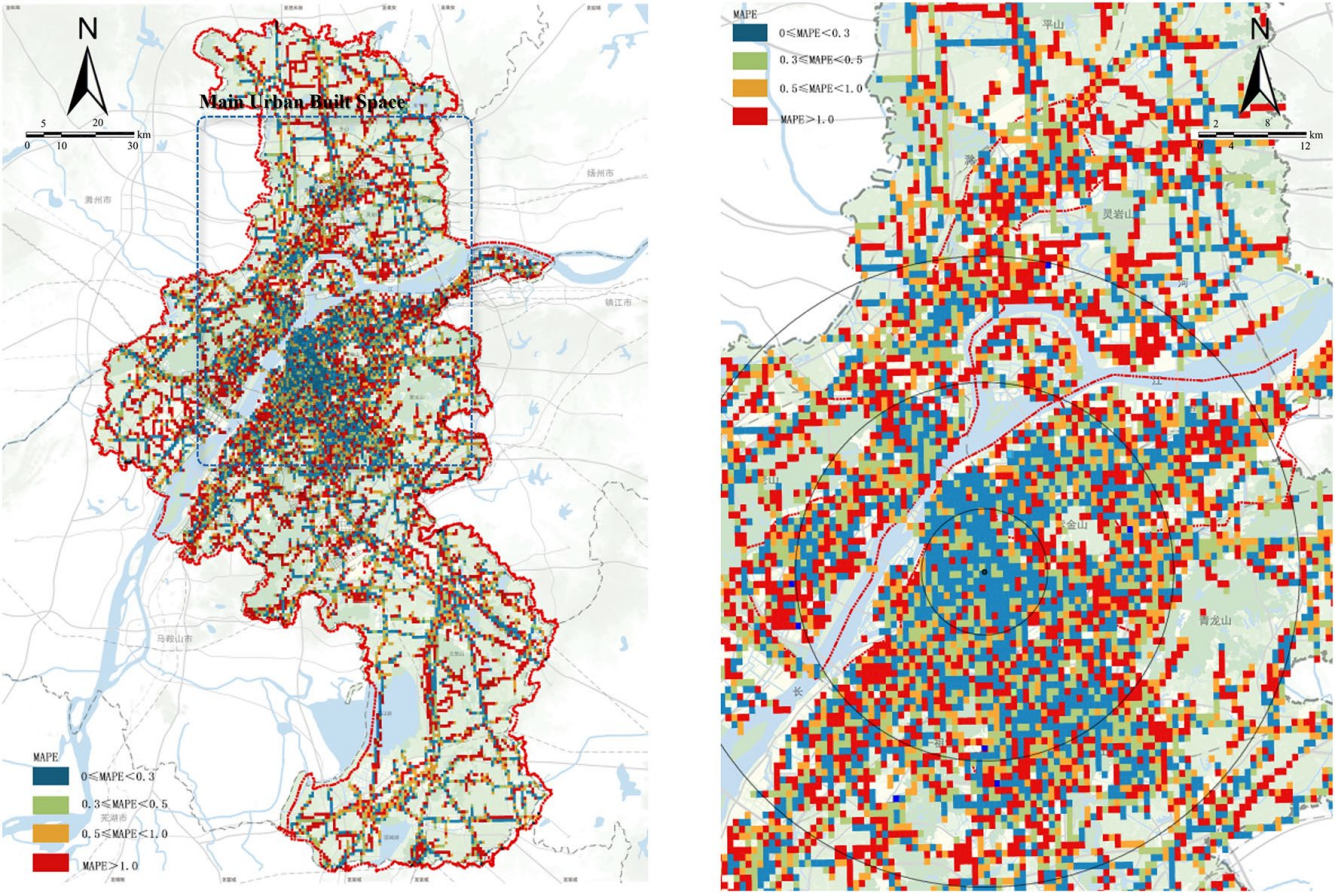
Spatial distribution of MAPE index (The left figure is MAPE is an overall urban space, while the right figure is MAPE is the main urban built space). *Source* Author made author made based on ESRI ArcGIS Pro Software (https://pro.arcgis.com/en/pro-app/latest/get-started/get-started.htm).

From the MAPE projection in urban space (Fig. 7), we can observe the spatial distribution pattern of model errors. It is found that in the core built area within 5 km from the main centre of the city, more than 90% of the spatial analysis units have the lowest MAPE, and the model fitting accuracy is the highest. In this scale, the model phase of this study is significantly higher than other similar studies at the level of the lowest error space coverage of the MAPE evaluation results^69,70^. Meantime, the model Within the central built area, the space units with higher fitting level are more evenly distributed, and the space units with lower fitting level are mostly linearly distributed along a certain road. Simultaneously, the new urban area under construction and development relatively has more errors than the completed areas.

In addition, from the perspective of population density in different spatial ranges, the model has a high fitting degree to the urban central area and the urban outer suburbs without centralized construction. These two types of areas correspond to the densely crowded area and the sparsely crowded area in the population density interval. However, the population density is still in the middle density stage of rapid development in the areas near the urban suburb and at the boundary of the built area under expansion and construction, and the model is less adaptable to these areas. As a result, the BEM prediction model features high simulation accuracy in the built space and certain new towns with relatively mature development. However, there is a large error in the prediction results of the model for the spatial range of the boundary of the urban suburban built area under expansion and construction.

### Correlation analysis between built environmental indicators and prediction errors

The Pearson correlation coefficient is used to analyse the correlation between the built environmental factors in the model and MAPE and observe the built environmental factors caused by model errors. It is found that the spatial capacity, land mixedness, accessibility, and business maturity have a positive impact on the accuracy of the model. The core and central space circles are advantageous over the outer suburbs in the above indicators which has confirmed the high accuracy of the core space circle and the main urban built space models^71^. In terms of land-use types, the urban space dominated by residential and public welfare service land is more accurate, while the urban area dominated by business, commercial, and industrial land is less accurate. Other built environmental factors do not have any significant correlation with the accuracy of the model (Table 4).

**Table 4:**
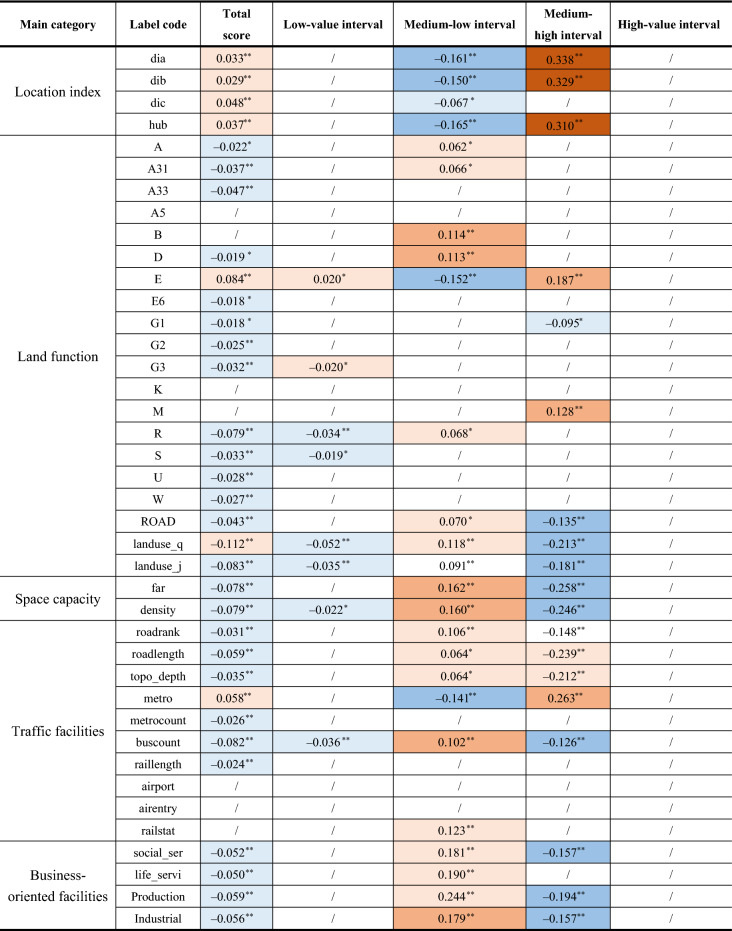
Correlation coefficients between average percentage error of classified agglomeration degree and built environmental indicators. *Source* Author made.

### Discussion on possible error reasons

According to the model prediction and evaluation results based on the above data set, the areas with the highest model fitting accuracy are the high-value and low-value areas of the urban population density, while the areas at the boundary of the built urban suburbs are characterized by low model accuracy. In terms of the prediction accuracy, it is further confirmed that there is a closer relationship between the population space–time density and the built environment in the high-value interval. From the conditions of the built environment, the model exhibits a low average MAPE for the prediction of the mature urban built space, and a low average MAE for the prediction of the decentralized urban construction areas. Therefore, the model can is adaptable for the prediction of the urban central space and the decentralized urban construction areas. Moreover, the urban central area is relatively well-developed, while the urban outer suburbs are relatively underdeveloped; thus, the built environment and population density are in a stable state in such two types of areas. Furthermore, the boundary area of the urban built suburbs is under rapid expansion and construction; thus, the spatial development and population activities have not been synchronized yet. However, in reality, the areas where the spatial–temporal distribution of urban population is not in agreement with the urban construction often appear in the new urban districts under expansion. The built environment in such areas changes rapidly, the relevant supporting facilities are often immature, and the population activities are more affected by economic and social factors; thus, the model is affected by larger fitting errors.

In addition, the difference of errors in different urban areas dominated by different functions shows that the model is more accurate for the urban areas dominated by residential and public welfare service land, but less accurate for the urban areas dominated by industrial and commercial land. In addition, the activity types and time rules of the population in residential and public welfare service land are relatively stable, while the activities are relatively complex and the time rules are weak in the industrial and commercial land which may result in the difference in the accuracy of the models for different types of land. Therefore, in the updated model optimization, the activity contents of industrial land and commercial business area shall be further subdivided by the type of industry, mode of production, and other relevant factors to improve the accuracy of the model.

## Conclusion

Considering the development trend of data-driven scientific paradigm and intelligent technology, this paper first discussed the characteristics and trends in the field of the current smart city research. Furthermore, against the background of new digital and intelligent technology, this paper has reviewed the existing methods in the field of urban population spatial–temporal behaviour prediction and simulation. Accordingly, through the summary and analysis of the interaction between urban built environment and urban population spatial–temporal behaviours, 40 categories of built environmental factors that have an impact on the population spatial–temporal behaviours are summarized. Furthermore, a model framework is proposed for the prediction of urban population spatial–temporal density distribution with urban built environment as a variable. Subsequently, the depth residual model in the convolutional neural network model is selected for modelling, training, and prediction, and correlation analysis and empirical test were used to evaluate the prediction model based on the test data set and analyse the possible causes of errors. In this study, the relevant algorithms in the field of computational vision were used to convert the built environment features into images, consider the spatial autocorrelation of the built environment, and use the residual neural network method in the convolution neural network to construct a prediction framework. The performance of the proposed prediction model is considerably better than that of the traditional regression algorithms and unvisualized machine learning algorithms.

Nonetheless, the behaviour–environment agent model (BEM) based on the interaction between built environment and population spatial–temporal behaviours proposed in this study still has many limitations. Firstly, the data dimension and accuracy need to be further improved. In the process of research, certain errors are found to result directly from the space–time deviation of the data; for example, the method of path finding and trajectory filling is used to compensate for the personal trajectory based on the base station in the study. Nonetheless, there are considerable errors in the identification of the population trajectory in the urban suburbs and other areas with fewer base stations. Moreover, in addition to the built environment, there are many external random factors that determine the human behaviours; thus, the predictive power is somewhat limited if the population is predicted only from the perspective of the built environment. In this regard, future research will further improve the factor indicators that have an impact on the population spatial–temporal behaviours from the urban spatial and social factors. Simultaneously, the weight of its impact on the population spatial–temporal behaviours shall be analysed in a quantitative manner, in order to improve the index basis of the prediction model and the accuracy of the prediction.

## Data description and availability

### Data introduction

The experimental data set used in this study consists of the data of mobile phone users within Nanjing City provided by China Mobile (A personal communication service provider company). The personal information data has been declassified and does not contain personal privacy information. This data set is subject to the spatial range of the Nanjing City with the data collected over 8 working days in November 2019. Moreover, the mobile phone users are more than 2 million every day; therefore, the mobile phone signaling data after cleaned can reflect the population overall behaviors in Nanjing. The cleaned mobile phone signaling data includes four fields: the user ID after declassification, location timestamp, corresponding base station number, and longitude and latitude coordinates of the corresponding base station. See Table 5 for sample data. Each user has 36 records on average; thus, the mobile phone signaling data set after being cleaned can better describe the individual's behaviors in a day.

**Table 5:** User’s mobile phone signaling data. *Source* Author made.

UserID	Station	Time	X	Y
000_705********	93,637***	201501109050323	118.8242**	31.226**
000_705********	93,637***	201501109070324	118.8242**	31.226**
000_705********	93,642***	201501109091024	118.7892**	32.011**
000_705********	…	…	…	…
000_705********	93,637***	201501109223521	118.8242**	31.226**

The built environment data used in this study are mainly sourced from the building, land use, road traffic, and business POI data. Among them, the land use, building, and road traffic data are authorized and provided by the planning management department (Natural Resources and Planning Bureau) of the study-involved city. The land use and building data are locally corrected according to field survey or open source data from Google satellite images. The business POI data are derived from the open source data provided by the geographic information network service provider, specifically the business data of the Nanjing City area of Amap in 2015 which contains spatial location, category, and other attribute information. See Table 6 for the source and basic information of the data related to the built environment.

**Table 6:** Sources and basic information of built environment data. *Source* Author made.

Data contents	Data format	Data source
Building contour and its surrounding area, including the building floor area, building floors, and other attribute information	shp	It is provided by the Nanjing Bureau of Planning and Natural Resources; the land use data and building data are locally corrected according to the field survey or Google satellite images
Plot contour and its surrounding area, including land property, land area, and other attribute information	shp	
Linear elements such as road centerlines at all levels, boundary lines of roads and railway alignment, of which the road centerline contains road grade attribute information; hub stations, urban rail transit stations, public transport stations, airport entrances and exits and other point-like elements	shp/csv	
Business POI data, including spatial location, category and other attribute information	csv	Baidu Map API

## Data Availability

The data that support the findings of this study are available from China Mobile Communications Group Co., Ltd, (http://it.10086.cn/indexc.html), and got data use permission from both China Mobile Company and Southeast University, China. But restrictions apply to the availability of these data, which were used under license for the current study, and so are not publicly available. Data are however available from the authors upon reasonable request and with permission of Both China Mobile Communications Group Co., Ltd and Southeast University, China. Any researcher or research team that would like to obtain the data mentioned in this article for research purposes should contact 400-16-10,086, or email yewuhezuo@chinamobile.com. Meantime, relevant research team also can connect yangjy_seu@163.com for more information and negotiate data and data usage requirements.
